# Statistical analysis for efficacy of tirofiban combined with ozagrel in the treatment of progressive cerebral infarction patients out of thrombolytic therapy time window

**DOI:** 10.6061/clinics/2021/e2728

**Published:** 2021-06-07

**Authors:** Huiying Zhang, Lei Zheng

**Affiliations:** I School of Statistics, ShanXi University of Finance & Economics, Taiyuan, Shanxi Province, 030006, China; II Department of Cardiovascular Medicine, ShanXi Bethune Hospital, Taiyuan, Shanxi Province, 030032, China

**Keywords:** Tirofiban, Ozagrel, Progressive Cerebral Infarction, Out of Thrombolytic Therapy Time Window

## Abstract

**OBJECTIVES:**

To investigate the safety and efficacy of combined tirofiban-ozagrel therapy for treating progressive stroke patients out of thrombolytic therapy time window.

**METHODS:**

This prospective, double-blind, randomized controlled study included 337 patients who had experienced an acute ischemic stroke between November 2017 and December 2018. All patients were randomized into three groups: 1) the tirofiban/ozagrel group (n=113), 2) the tirofiban group (n=110), and 3) the ozagrel group (n=114). The platelet aggregation (PAG), thrombin time (TT), prothrombin time (PT), activated partial thromboplastin time (APTT), and fibrinogen (FIB) levels in the patients from these groups were evaluated before starting treatment and then, at 24h, 7 days, and 14 days after treatment. The National Institutes of Health Stroke Scale (NIHSS) scores were evaluated before treatment and then, 24h, 1 week, 2 weeks, and 4 weeks after treatment. The Barthel Index (BI) score was used to measure safety, and the modified Rankin scale (mRS) was used to evaluate disability following 3 months of treatment. The risk factors affecting clinical outcomes were analyzed using logistic multivariate regression.

**RESULTS:**

The mean NIHSS score for all the patients was 13.17±3.13 before treatment, and no significant difference between the basic clinical parameters of the three patient groups was found. Following treatment, both PAG and FIB were significantly reduced compared with the baseline (*p*<0.05). The levels of PAG and FIB in the tirofiban/ozagrel group were significantly lower than those in the tirofiban and ozagrel groups at 24h and 7 days after treatment (*p*<0.05). The NIHSS score decreased significantly in all treatment groups (*p*<0.05). The tirofiban/ozagrel NIHSS scores were significantly lower than that of the tirofiban and ozagrel groups at 24h, 1 week, and 2 weeks post initiation (*p*<0.05 for all). There were no significant differences in the BI and mRS scores or the intracranial hemorrhage rates; further, age, sex, Trial of ORG 10172 in acute stroke treatment (TOAST) type, baseline NIHSS and 24-h NIHSS scores, baseline thrombus-related factors, and treatment methods were shown to not be independent risk factors for clinical outcomes.

**CONCLUSION:**

The combination of tirofiban and ozagrel, as well as monotherapy with either tirofiban or ozagrel, transiently improves the neural function of patients and reduces platelet aggregation and fibrinogen formation in the first 4 weeks following a stroke event; additionally, none of these treatments increased the risk for hemorrhage in these progressive stroke patients over a 3-month period.

## INTRODUCTION

There are almost 7 million ischemic stroke-like incidents every year and around 1.5-2.0 million new stroke cases every year in China (1-3). In general, thrombolytic therapy is the most effective treatment strategy for acute ischemic stroke (4,5). However, because of the short time window for thrombolytic therapy, thrombolysis cannot be used in a large number of patients (6). Under these conditions, antiplatelet treatment is needed (7,8).

Tirofiban is a reversible non-peptide platelet surface glycoprotein (GP) IIB/III receptor antagonist that is widely used in the treatment of various cardiovascular diseases, and also in the prevention of coronary heart disease (9,10). Recent studies have also suggested that tirofiban could be used to prevent reocclusion and restenosis following thrombolysis or mechanical thrombectomy and may improve clinical outcomes in these patients (11-13). However, studies describing the application of tirofiban for treating patients outside the thrombolytic therapy time window are rare. Ozagrel, a thromboxane synthetase (TXA2) inhibitor, is routinely used in the treatment of ischemic stroke (14). However, there are still relatively few clinical studies describing the efficacy and safety of ozagrel in the treatment of stroke patients.

In this study, we performed a prospective double-blind randomized controlled trial to investigate the efficacy and safety of using a combined tirofiban/ozagrel therapeutic strategy in the treatment of progressive stroke patients who were outside the thrombolytic therapy time window. This study provides more clinical evidence for the use of tirofiban and ozagrel in the treatment of stroke patients.

## MATERIALS AND METHODS

### Patients and treatment

This prospective, double-blind, randomized controlled study included 337 patients with acute ischemic stroke who visited ShanXi Bethune Hospital between November 2017 and December 2018. All patients who met the inclusion criteria were enrolled consecutively. The inclusion criteria were as follows: 1) all patients who were sent to the hospital within 72h of their stroke; 2) all patients diagnosed with ischemic stroke with no previous stroke history detected by imaging methods such as computerized tomography (CT) or nuclear magnetic resonance (MRI); 3) progressive cerebral infarction was defined as including the neurological deficits that did not show any improvement and continued to progress after 1 week of treatment with aspirin (100 mg/d) and clopidogrel bisulfate (75 mg/d), and a National Institutes of Health Stroke Scale (NIHSS) score that increased by at least 2 points; 4) patients with 4≤NIHSS scores≤18; and 5) all patients had to fall outside the time window for thrombolytic therapy when sent to the hospital. The exclusion criteria included: 1) patients with a history of stroke; 2) hemorrhagic stroke; 3) patients who had taken anticoagulants within 3 months of the study; 4) patients with hereditary or acquired bleeding constitution or hemorrhagic diseases or those who underwent surgeries within 3 months of the study; 5) patients with blood sugar levels <60 mg/dl or with platelet counts <10^6^/mm^3^; and 6) patients with severe renal or liver dysfunction. This study was approved by the ethics committee at the ShanXi Bethune Hospital, institutional review board approval number: 2017053.

For the treatment of the patients, all patients were randomized into three groups using a computer-generated list. Because the study was double-blind, only the study designer knew the patient grouping, and all reagents and medications were assigned by a third department. All patients were divided into three groups: 1) the tirofiban/ozagrel group (n=113), 2) the tirofiban group (n=110), and 3) the ozagrel group (n=114). For the tirofiban group, 12.5 mg of tirofiban hydrochloride (Yuanda pharmaceutical China Co., Ltd., No.: H20041165) was dissolved in 250 ml of normal saline and intravenously injected at a dose of 0.4 μg/kg/min for 30 min and then, at a dose of 0.1 μg/kg/min over the next 47.5h. For the ozagrel group, 40 mg of ozagrel (Changchun Haobang Pharmaceutical Co., Ltd, No.: H20031038) was dissolved in 250 ml of normal saline and the patients received intravenous injections twice a day. The tirofiban/ozagrel group received both tirofiban and ozagrel as described above. Both the tirofiban and ozagrel group received their therapeutic treatment and 250 ml of normal saline (as a placebo). Treatment for all groups lasted 14 days. After treatment, all patients received the usual anti-thrombotic therapy, and CT or MRI was conducted after 24h and 7 days of treatment.

### Measurement of thrombus-related factors

After treatment, the platelet aggregation (PAG), thrombin time (TT), prothrombin time (PT), activated partial thromboplastin time (APTT), and fibrinogen (FIB) levels were measured using a Sysmex CS-5100 automatic coagulation analyzer (Sysmex Medical Electronics Co., Kobe, Japan); the samples were evaluated before starting the treatment and at 24h, 7 days, and 14 days after treatment.

### Data collection

Demographic data such as age, sex, and clinical characteristics, including complications and medication, were also recorded. The NIHSS scores were evaluated before treatment and at 24h, 1 week, 2 weeks, and 4 weeks after treatment. The onset to treatment time (OTT) was defined as the time between stroke onset and the start of treatment. Hemorrhagic transformation was measured by CT or MRI after treatment according to the European Cooperative Acute Stroke Study (ECASS II) definitions (15). The Barthel Index (BI) score was used to measure safety, and the modified Rankin scale (mRS) was used to measure disability after 3 months of treatment. All patients were followed-up for 3 months as a part of this study.

### Statistical analysis

Continuous data were expressed as the mean±standard deviations. The chi-square test was used to compare discrete variables and incidence rates, while comparisons between two groups were performed using the Student’s t-test. The logistic analysis comprised logistic multivariate regression using a logistic regression model fixed using a stepwise method and was used to identify the factors with a direct impact on the clinical outcome. Statistical significance was set at *p*<0.05. All calculations were performed using the SPSS 22.0 software (SPSS Inc., Chicago, IL, USA).

## RESULTS

### Patient characteristics

This study enrolled 337 progressive ischemic stroke patients who were divided into three groups: the tirofiban/ozagrel group (mean age, 60.29±11.20 years; male to female ratio, 62:51), the tirofiban group (mean age, 60.01±10.07 years; male to female ratio, 59:51), and the ozagrel group (mean age, 61.41±10.95 years; male to female ratio, 65:49). Among all the patients enrolled in this study, 318 were conscious, 14 were unconscious, and 5 were drowsy. Hemiplegia of the left limbs was identified in 172 patients and hemiplegia of the right limbs was identified in 165 patients. A total of 70 patients were aphasic and 124 patients experienced hypalgesia or loss of superficial limb pain. The mean NIHSS score of all patients was 13.17±3.13 before the study; there were no significant differences between the basic clinical characteristics of the patients from these groups (Table 1).

### Changes in thrombus-related factors in each group

We then evaluated the efficacy of each of the treatment strategies via the independent examinations of various thrombus-related factors before starting the treatment and then, at 24h, 7 days, and 14 days after the treatment. The results showed that both the PAG and FIB values were significantly reduced in the treatment groups, compared with the baseline values (*p*<0.05, Table 2). The levels of PAG and FIB in the tirofiban/ozagrel group were significantly lower than those in the tirofiban and ozagrel groups at 24h and 7 days after treatment (*p*<0.05). No significant differences were found for the other factors.

### Dynamic changes in the NIHSS scores of the patients

**Figure 1:**
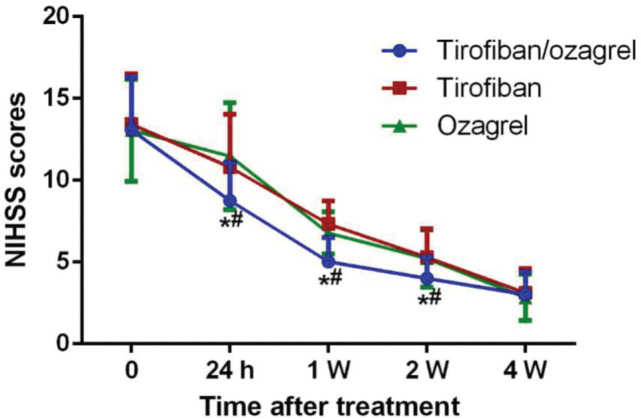
NIHSS scores of the patients from the different groups. *p<0.05 *versus* the Tirofiban group; ^#^ *p*<0.05 *versus* the ozagrel group.

The NIHSS scores were measured before treatment and then, at 24h, 1 week, 2 weeks, and 4 weeks after treatment. The NIHSS scores in all groups decreased gradually following treatment. At 24h, 1 week, and 2 weeks post treatment, the NIHSS scores in the tirofiban/ozagrel group were shown to be the most significantly reduced compared with the baseline (*p*<0.05); the NIHSS scores in both the individual treatment groups showed no significant difference at 4 weeks (Figure 1). These results suggest that both tirofiban and ozagrel may improve neural function in progressive stroke patients, and that the combination of tirofiban and ozagrel may facilitate this process.

### BI and mRS scores and the incidence of complications

**Figure 2:**
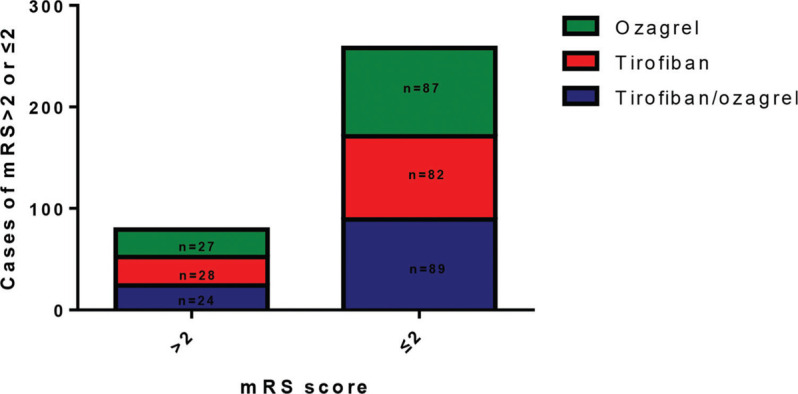
mRS scores for patients in different groups.

We also evaluated the BI and mRS scores for these patients at 3 months after treatment and recorded any complications. As shown in Table 3, no significant differences were found between the BI and mRS scores of the patients in the different groups; 258 subjects showed mRS scores of ≤2 and 79 subjects showed mRS scores of >2 (Figure 2). No mortality or severe side effects were observed. Intracranial hemorrhage was found only in 11 subjects (9.73%) in the tirofiban/ozagrel group, 9 subjects (8.18%) in the tirofiban group, and 10 subjects (8.77%) in the ozagrel group, with none showing hemorrhage and requiring blood transfusion.

### Logistic regression for identifying risk factors for disability in stroke patients

Finally, we used logistic regression analysis to evaluate the risk factors for disability in the patients. As mentioned above, we used mRS to measure disability; an mRS of >2 was defined as a disability. Age, sex, Trial of ORG 10172 in acute stroke treatment (TOAST) type, baseline NIHSS and 24-h NIHSS scores, baseline thrombus-related factors, and treatment methods were evaluated as risk factors. However, none of these factors were shown to be independent risk factors for disability (Table 4).

## DISCUSSION

Despite numerous studies on, and treatment methods for, stroke, the treatment of stroke patients outside the thrombolysis time window remains a clinical challenge (16-19). In this study, we report the efficacy of combined tirofiban/ozagrel therapy in the treatment of patients with progressive cerebral infarction who were admitted at the hospital outside the therapeutic window for thrombolytic therapy. We found that the combination of tirofiban and ozagrel, as well as monotherapy with either tirofiban or ozagrel, could improve the neural function of the patients and reduce platelet aggregation and fibrinogen formation; thus, these therapeutic modalities could be used in the treatment of progressive stroke patients. In addition, the combination of tirofiban and ozagrel may promote patient recovery; this combination did not increase the risk of hemorrhage.

Tirofiban is primarily used in the prevention of heart ischemic diseases, such as myocardial infarction and unstable angina. However, recent studies have also demonstrated that tirofiban can be used for the treatment of ischemic stroke. It was reported that treatment with tirofiban after thrombolysis using alteplase improved the NIHSS scores of the patients and reduce the reocclusion rate (20). However, controversial results have been reported with regard to the risk of tirofiban-induced hemorrhage. Lee et al. reported that tirofiban did not increase the risk of hemorrhage after bridging antiplatelet therapy (21). However, another study observed the opposite result: treatment with tirofiban increased the risk of symptomatic intracranial hemorrhage by 2.9-fold in patients following thrombectomy (22). These results indicate that the application of tirofiban should be carefully evaluated for hemorrhage risk.

In addition to treatment after thrombolysis or mechanical thrombectomy, several recent studies have also found that tirofiban could be used for patients who were not able to undergo thrombolytic therapy or for whom mechanical thrombectomy failed. Seo et al. reported that adjuvant tirofiban injection could be used for acute stroke patients for whom mechanical thrombectomy failed, with a recanalization rate of 77.7% being observed after the intra-arterial injection of tirofiban and subsequent Solitaire thrombectomy (23). Another study demonstrated that tirofiban could be used for patients experiencing an acute ischemic stroke without arterial occlusion who were outside the window for thrombolytic therapy and that this treatment enhanced the 3-month outcomes, compared with those of patients in the control group (24). A more recent study also showed that intra-arterial tirofiban administration could be used for patients after unsuccessful mechanical thrombectomy, with no increase in symptomatic intracranial hemorrhage risk (25). Based on these data, tirofiban may be more appropriate and safer for the treatment of patients for whom thrombolytic therapy and mechanical thrombectomy cannot be used. In this study, we also reported that tirofiban improved the neural function and reduced platelet aggregation and fibrinogen formation in progressive stroke patients.

There are very few clinical studies describing the use of ozagrel in the treatment of stroke. One Japanese study found that ozagrel did not increase the risk of hemorrhagic complications in patients with atherothrombotic stroke or lacunar infarction; however, it did not significantly improve functional outcomes (26). Another study showed that ozagrel and argatroban had similar efficacies in the treatment of acute non-cardioembolic stroke, regardless of the use of edaravone (27); further, in an animal study, the authors found that pretreatment with ozagrel may show neuroprotective effects in rats with stroke (28). However, there is little information regarding the application of ozagrel for treating stroke, indicating a clear need for further evaluations of its application in the treatment of ischemic stroke. Our study showed that both ozagrel and a combination of ozagrel and tirofiban could be used for the treatment of patients with progressive stroke without increasing the risk of hemorrhage.

This study does have some limitations. First, the sample size was limited. Second, we did not investigate the long-term efficacy of these two drugs. Finally, there was no ‘standard treatment’ control, and the outcome was only assessed within the first 3 months. Thus, further extensive studies that also take these aspects into consideration are needed to clarify the role of ozagrel and tirofiban in the management of acute ischemic stroke.

In conclusion, this prospective double-blind randomized controlled study found that the combination of tirofiban and ozagrel, as well as monotherapy with tirofiban or ozagrel, transiently improved the neural function and reduced platelet aggregation and fibrinogen formation in progressive stroke patients in the first 4 weeks of therapy, without increasing the risk of hemorrhage in the 3 months following the clinical event. This study may provide more clinical evidence for the application of tirofiban and ozagrel in patients who fall outside the time window for thrombolytic therapy.

## AUTHOR CONTRIBUTIONS

Each author made important scientific contributions to the study and assisted with the drafting or revision of the manuscript.

## Figures and Tables

**Table 1:** Demographic and baseline characteristics of the total study cohort.

Variables	Tirofiban/ozagrel group, n=113	Tirofiban group, n=110	Ozagrel group, n=114	*p-*value*
Age, year	60.29±11.20	59.96±9.93	61.57±11.13	0.494
BMI, kg/m^2^	22.92±2.37	22.71±2.21	23.11±2.30	0.418
Sex, male: female	62: 51	59:51	65:49	0.888
Risk factors, n (%)				0.928
Hypertension	48 (42.48)	42 (38.18)	44 (38.60)	
Diabetes	35 (30.97)	37 (33.64)	31 (27.19)	
Current smoker	57 (50.44)	59 (53.64)	51 (44.74)	
History of Coronary heart disease	21 (18.58)	15 (13.64)	24 (21.05)	
History of Atrial fibrillation	18 (15.93)	22 (20.00)	21 (18.42)	
Family history of Stroke	20 (17.70)	17 (15.45)	23 (20.18)	
TOAST type, n (%)				0.663
Cardiogenic embolism	39 (34.51)	35 (31.82)	41 (35.96)	
Large-artery Atherosclerosis	35 (30.97)	32 (29.09)	37 (32.46)	
Small-artery occlusion	31 (27.43)	29 (26.36)	30 (26.32)	
Others	8 (7.08)	14 (12.73)	6 (5.26)	
Baseline NIHSS	13.07±3.20	13.45±3.12	12.99±3.09	0.837
Onset to treatment time (OTT), h	40.23±20.77	39.30±19.80	40.90±19.56	0.506

* The chi-square test was used to compare the count values and incidence rates, and comparisons between two groups were performed using a Student’s t-test. NIHSS: National Institutes of Health Stroke Scale; TOAST: Trial of ORG 10172 in Acute Stroke Treatment.

**Table 2:** Changes in various thrombus-related factors in response to the various treatments.

Variables	Treatment	Before	24h	7 days	14 days
PAG, %	Tirofiban/ozagrel	87.73±12.09	75.20±9.16^b^,^c^	63.97±10.65^b^,^c^	57.29±6.90
	Tirofiban	91.03±11.98	82.71±10.23^a^,^c^	75.47±10.75^a^,^c^	57.61±7.02
	Ozagrel	87.99±12.49	82.65±9.96^a^,^b^	75.61±11.35^a^,^b^	56.78±7.51
TT, s	Tirofiban/ozagrel	18.06±2.38	18.18±2.28	17.97±2.31	18.12±2.19
	Tirofiban	18.03±2.44	18.19±2.27	17.85±2.22	17.65±2.21
	Ozagrel	17.75±2.30	18.07±2.34	18.12±2.23	17.96±2.41
PT, s	Tirofiban/ozagrel	12.97±1.17	12.99±1.17	13.00±1.23	13.06±1.12
	Tirofiban	12.97±1.12	12.95±1.18	12.98±1.27	12.94±1.11
	Ozagrel	13.1±1.10	13.05±1.18	13.10±1.17	12.98±1.19
APTT, s	Tirofiban/ozagrel	28.02±2.29	28.26±2.26	27.90±2.31	27.87±2.18
	Tirofiban	28.15±2.13	28.51±2.28	27.98±2.33	27.99±2.42
	Ozagrel	27.93±2.46	28.12±2.15	27.84±2.37	28.03±2.18
FIB, g/L	Tirofiban/ozagrel	3.95±0.089	3.65±0.056^b^,^c^	3.37±0.097^b^,^c^	3.28±0.11
	Tirofiban	3.95±0.087	3.81±0.075^a^,^c^	3.46±0.097^a^,^c^	3.27±0.097
	Ozagrel	3.95±0.084	3.81±0.073^a^,^b^	3.48±0.108^a^,^b^	3.26±0.098

a *p*<0.05, *versus* the tirofiban/ozagrel group

b *p*<0.05 *versus* the tirofiban group

c *p*<0.05, *versus* the ozagrel group. PAG, platelet aggregation; TT, thrombin time; PT, prothrombin time; APTT, activated partial thromboplastin time; and FIB, fibrinogen levels. The chi-square test was used to compare the values for these variables. Comparisons between two groups were performed using the Student’s t-test.

**Table 3:** The BI and mRS values and the incidence of intercranial hemorrhage in each group.

Variables	Tirofiban/ozagrel group, n=113	Tirofiban group, n=110	Ozagrel group, n=114	*p*-value
BI	78.83±8.75	80.60±9.54	79.55±9.30	0.355
mRS	1.64±1.10	1.56±1.21	1.49±1.14	0.602
Intracranial hemorrhage, n (%)	11 (9.73)	9 (8.18)	10 (8.77)	0.927

*Comparison between two groups was performed using the Student’s t-test.

**Table 4:** Logistic regression analysis of clinical risk factors affecting long-term disability in stroke patients.

	Wald	Odds ratio	95% CI	*p-*value
Age	0.267	0.007	1.006 (0.981-1.032)	0.592
Sex	0.402	0.170	1.211 (0.707-2.070)	0.493
TOAST type	1.634	0.174	1.171 (0.891-1.539)	0.182
Baseline NIHSS	2.418	-0.067	0.929 (0.851-1.015)	0.110
24h NIHSS	1.110	-0.048	0.945 (0.864-1.034)	0.281
Baseline PAG	3.775	0.021	1.020 (0.998-1.043)	0.054
24 PAG	0.001	0.000	1.001 (0.972-1.029)	0.945
Baseline TT	1.587	-0.071	0.934 (0.834-1.046)	0.208
24h TT	0.052	0.014	1.013 (0.898-1.142)	0.833
Baseline PT	0.371	0.073	1.087 (0.854-1.383)	0.525
24h PT	0.001	-0.003	0.994 (0.789-1.254)	0.965
Baseline APTT	0.539	-0.044	0.966 (0.856-1.089)	0.499
24h APTT	0.415	0.040	1.048 (0.927-1.185)	0.445
Baseline FIB	2.346	2.383	11.45 (0.494-264.997)	0.100
24h FIB	0.010	0.193	0.654 (0.013-32.787)	0.832
Treatment with tirofiban/ozagrel	0.244	0.226	1.341 (0.461-3.895)	0.589
